# Ni-SiO_2_ Catalysts for the Carbon Dioxide Reforming of Methane: Varying Support Properties by Flame Spray Pyrolysis

**DOI:** 10.3390/molecules20034594

**Published:** 2015-03-12

**Authors:** Emma C. Lovell, Jason Scott, Rose Amal

**Affiliations:** School of Chemical Engineering, The University of New South Wales, Sydney, NSW 2052, Australia; E-Mails: e.lovell@unsw.edu.au (E.C.L.); r.amal@unsw.edu.au (R.A.)

**Keywords:** nickel catalysts, carbon dioxide reforming, flame spray pyrolysis, silica

## Abstract

Silica particles were prepared by flame spray pyrolysis (FSP) as a support for nickel catalysts. The impact of precursor feed rate (3, 5 and 7 mL/min) during FSP on the silica characteristics and the ensuing effect on catalytic performance for the carbon dioxide, or dry, reforming of methane (DRM) was probed. Increasing the precursor feed rate: (i) progressively lowered the silica surface area from ≈340 m^2^/g to ≈240 m^2^/g; (ii) altered the silanol groups on the silica surface; and (iii) introduced residual carbon-based surface species to the sample at the highest feed rate. The variations in silica properties altered the (5 wt %) nickel deposit characteristics which in turn impacted on the DRM reaction. As the silica surface area increased, the nickel dispersion increased which improved catalyst performance. The residual carbon-based species also appeared to improve nickel dispersion, and in turn catalyst activity, although not to the same extent as the change in silica surface area. The findings illustrate both the importance of silica support characteristics on the catalytic performance of nickel for the DRM reaction and the capacity for using FSP to control these characteristics.

## 1. Introduction

The carbon dioxide (dry) reforming of methane (DRM) has been extensively investigated in recent decades. The capacity for the reaction to consume the most destructive greenhouse gases (CO_2_ and CH_4_) to produce synthesis gas (H_2_ and CO; Equation (1)) make it a possible solution to some of the key anthropogenic issues facing society. Synthesis gas (syngas) can be used to produce liquid fuels such as methanol or diesel by the Fischer-Tropsch reaction and thus allow a transition to a more sustainable future.

(1) $$CH_{4}+CO_{2}\to 2H_{2}+2CO$$

For catalysts to be utilised on an industrial scale they need to be active, selective and stable as well as economically viable. Thus, while noble metal based catalysts are more active and stable with reduced carbon formation [1,2] their costs limit commercial application. Ni provides a viable alternative from an economic perspective which promotes its favoritism as a catalyst in some cases.

Beyond the catalyst components, a manner of producing catalysts that is feasible on an industrial scale is essential. Flame spray pyrolysis (FSP) is a high-production-rate, reproducible method for fabricating nanoparticles [3,4,5]. FSP combusts low-cost organometallic precursors to produce mixed metal oxide powders, generally <200 nm in diameter. Parameters, such as precursor concentration and feed rate can be tuned to vary particle size (and therefore surface area) making FSP a facile means to produce catalyst supports with controlled characteristics. Recent research has focused on modifying support properties to improve Ni catalysts for the DRM, particularly strategies to decrease carbon formation and ultimately increase catalyst stability. The use of highly structured, mesoporous supports facilitate small Ni size with high dispersion which ultimately results in a more active, stable catalyst [6,7]. Moreover, the incorporation of high oxygen mobility and/or basic supports, such as ceria, has been proven to decrease coking and improve catalytic performance [8]. FSP allows for one step-production of single and mixed-metal oxides that can be utilised as supports for the DRM. Silica is a regular choice as a support material as it can possess a high surface area and is generally considered to be inert [9,10]. SiO_2_ also has the benefits of being abundant and cheap with silica produced using FSP having reported surface areas up to 400 m^2^/g [4]. As supports fashioned by FSP are yet to be considered as catalysts for many reactions, including the DRM, studies being performed on a relatively inert support will provide a clearer understanding of the impact of flame conditions on catalytic performance, as opposed to using a more complicated mixed-metal oxide system. Varying support properties will have a significant impact on the size and distribution of active sites on a catalyst surface. In the context of the DRM, Ni size and metal-support interaction influences catalyst stability (most significant at >10 nm) [11,12,13]. During impregnation the surface properties of silica will impact dispersion and size of the deposited catalytic metal. For instance, it is well known that there are two types of silanol groups on silica surfaces; H–bonded Si–OH and isolated (non H-bonded) Si–OH [14,15,16]. Qu *et al.* studied Ag impregnation on SiO_2_ and found that varying silanol groups by calcination significantly altered the active site dispersion and metal-support interaction [17]. This resulted in increased activity and selectivity for the low temperature catalytic oxidation of carbon monoxide [17].

In this work FSP was utilised to synthesise silica at varying precursor feed rates (3, 5 and 7 mL/min). Whilst recent research has demonstrated the importance of silica surfaces during impregnation [17,18] the impact of varying flame conditions during FSP on the surface chemistry of produced silica has, to the best of the authors’ knowledge, not been reported. This effect of the varying flame parameters on the neat silica characteristics was assessed by N_2_ adsorption/desorption isotherms (and Brunauer-Emmett-Teller, BET surface area), Fourier transform infrared spectroscopy (FTIR), and transmission electron microscopy (TEM). The silica samples were impregnated with 5 wt % Ni to obtain sufficient conversion whilst minimising carbon formation. The NiO deposit properties were examined by X-ray diffraction (XRD), BET surface area, TEM and H_2_ temperature programmed reducibility (H_2_-TPR). The samples were activity tested and the impact of the varying flame properties on the Ni species present as well as the activity and stability of the prepared samples was evaluated.

## 2. Results and Discussion

**Figure 1:**
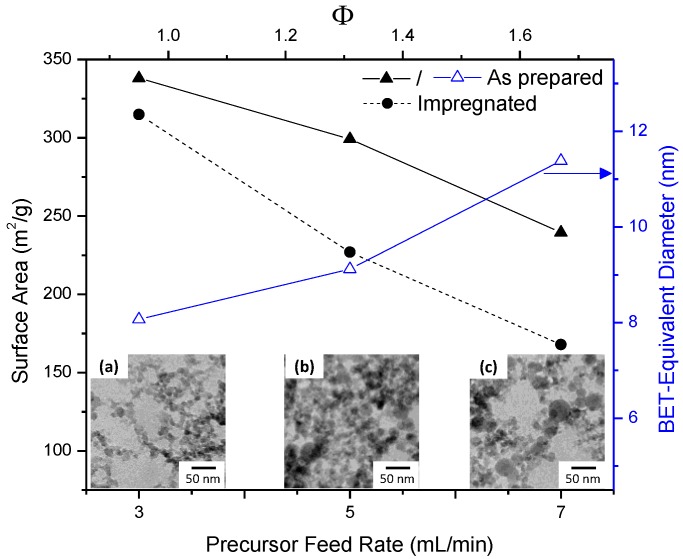
Impact of precursor feed rate and fuel equivalence ratio (Φ) on specific surface area and Brunauer-Emmett-Teller (BET)-equivalent diameter of flame spray pyrolysis (FSP)-produced silica; as prepared, and after 5% Ni impregnation and calcination. Included are TEM micrographs of FSP (**a**) 3, (**b**) 5 and (**c**) 7-SiO_2_.

Figure 1 shows the impact of precursor feed on silica surface area as well as BET equivalent diameter (calculated by d_BET_ = 6/(SSA × ρ_silica_) where SSA is the specific surface area of the prepared particles in cm^2^/g and ρ_silica_ is the density of silica, taken as 2.2 g/cm^3^ [5]) for the 3, 5 and 7 mL/min fed FSP silica, hereafter referred to as 3, 5 and 7-SiO_2_, respectively. Increasing precursor feed rate from 3 to 7 mL/min resulted in a decrease in surface area from ~340 to 240 m^2^/g. A higher precursor feed rate means that more fuel is being fed to the flame resulting in a hotter flame (evidenced by the increasing flame height from ~6.5 to 9 cm). Additionally, a higher precursor feed rate means a higher concentration of silica within the flame, in turn promoting sintering (by increasing the likelihood of collisions). By increasing the fuel content and silica concentration within the flame the particle growth rate and sintering is encouraged thus resulting in larger particles [5].

Figure 1a–c shows the TEM images of FSP silica at differing feed rates. The micrographs support the BET surface areas; showing that the average particle size grows with increasing feed rate. As expected, FSP-made silica is spherical, exhibiting differing degrees of sintering and hard agglomeration. Higher feed rates show larger, more spherical particles due to a higher degree of sintering (Figure 1c) as well as less homogenous particle size distributions with 7-SiO_2_ possessing particles up to 40 nm in diameter.

**Figure 2:**
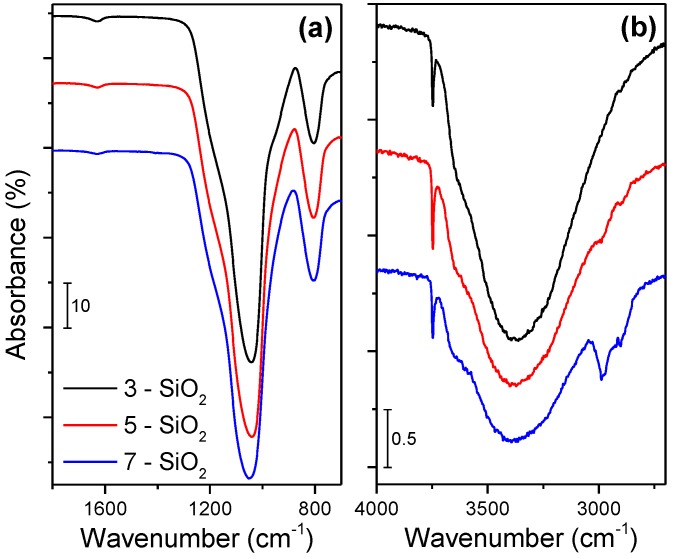
FTIR spectra of as prepared 3, 5 and 7-SiO_2_ showing the (**a**) 1800–700 cm^−1^ and (**b**) 4000–2700 cm^−1^ wavenumber regions.

To understand the impact of varying FSP conditions on the surface properties of the silica, FTIR was utilised. Figure 2 compares the FTIR spectra of the FSP silica from differing feed rates. With the exception of the band present at approximately 1630 cm^−1^ (which corresponds to free water most likely adsorbed on the silica surface [19]), Figure 2a depicts the adsorption peaks arising from the silica. The absorption band centered at 1046 cm^−1^ represents symmetric Si–O–Si with a shoulder at 970 cm^−1^ being asymmetric Si–OH and 803 cm^−1^ symmetric Si–O [20,21,22].

The absorption bands do not show any significant differences in the nature of the silica when prepared at different feed rates. In contrast, the FTIR spectra in Figure 2b display some variances in the surface species present on the silica. The broad peak near 3387 cm^−1^ corresponds to hydrogen bonded –OH groups (which can be attributed to H–bonded H_2_O, H–bonded –OH vibrations of residual alcohol and H–bonded Si–OH [23]). The sharp band evident at 3749 cm^−1^ corresponds to isolated silanol groups [23]. While the resolution makes it difficult to quantify the ratio of H–bonded to isolated –OH groups on the silica surface there is evidence of some variations. The percentage of isolated –OH groups to H–bonded –OH groups (normalised to the maximum Si–O–Si peak) increases with increasing precursor feed rate from 0.9%, 1.8% to 2.1% with 3, 5 and 7 mL/min, respectively.

The fuel equivalence ratio (Φ), which is a numerical representation of the oxygen supply to the flame [24], is displayed in Figure 1 (upper y-axis). A Φ value > 1 indicates that the flame is oxygen deficient. Irrespective of the precursor feed rate, a consistent oxygen flow is supplied to flame, and thus a higher precursor feed rate results in a more oxygen deficient flame. As expected, the 7 mL/min precursor flow rate provides the most oxygen deficient flame with an equivalence ratio of approximately 1.7. The impact of the elevated Φ is illustrated in Figure 2 with the presence of distinct C–H stretches around 2780–2880 cm^−1^ for 7-SiO_2_ [18,20,25]. The C–H bonding presence implies incomplete precursor combustion which results in residual C–H groups (2980 cm^−1^ is CH_3_, 2930 cm^−1^ is CH_2_ [20]) residing on the silica surface. There is some evidence of a minor C–H bonding presence on 5-SiO_2_ (Φ = 1.3) while the 3-SiO_2_ (Φ = 0.95) does not possess any evidence of C–H surface species. It should be noted that FTIR was also performed on all the samples after impregnation and calcination. No C–H adsorption bands were observed in this instance, indicating that calcination is sufficient for oxidising the residual carbon species.

### 2.1. Nickel Properties

Following impregnation with 5% Ni, and calcination TEM, XRD and H_2_-TPR were used to characterise the nature of the NiO deposits. After impregnation and calcination the surface area of the samples decreased by approximately 7%–30% (Figure 1). As FSP synthesised materials are non-porous the decrease in surface area can be attributed to sintering due to heat treatment of the particles.

**Figure 3:**
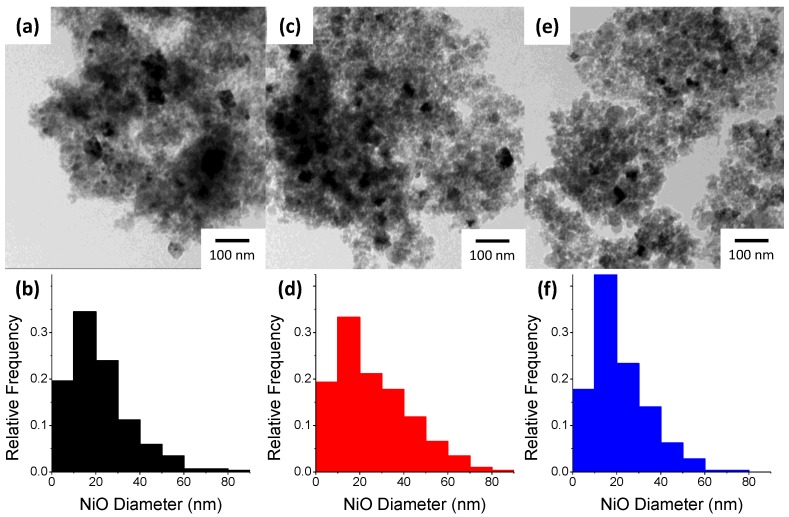
TEM images and corresponding NiO size distribution of (**a**,**b**) 3 (**c**,**d**) 5 and (**e**,**f**) 7-SiO_2_ after impregnation with 5% Ni and calcination.

Figure 3 displays the TEM micrographs and corresponding NiO deposit size distributions on the varying silica supports with a minimum of 300 measured deposits. The average measured deposit sizes are shown in Table 1. From the results it is evident that 3-SiO_2_ has the smallest NiO deposit size. The 3-SiO_2_ fed FSP silica has the smallest SiO_2_ particle size and therefore has the largest area for the Ni to disperse across during impregnation, thus resulting in the smallest deposits. Our previous findings have shown the impacts of higher surface area supports during Ni impregnation, whereby a greater surface area support facilitated a higher Ni dispersion [6]. Interestingly, the increasing average NiO deposit size does not follow the decrease in SiO_2_ surface area for the 5 and 7-SiO_2_. 7-SiO_2_ has a slightly smaller average NiO deposit size than the 5-SiO_2_ with the narrowest particle size distribution of all samples. 5-SiO_2_ shows the broadest NiO size distribution along with the highest average deposit size.

**Table 1:** Summary of Ni properties of Ni-SiO_2_ with silica being prepared by FSP at varying feed rates.

Silica Feed Rate (mL/min)	Actual Ni Loading (%) ^a^	NiO Size (nm)	
		TEM (σ) ^b^	XRD ^c^
3	4.4	21.7 (13.9)	13.3
5	4.3	25.9 (16.1)	13.8
7	4.4	24.9 (12.3)	13.2

^a^ Actual Ni loading determine from ICP-OES; ^b^ Average deposit size from at least 300 particles, with σ being the standard deviation of the sample; ^c^ NiO crystal size determined from (200) reflection from XRD profiles.

**Figure 4:**
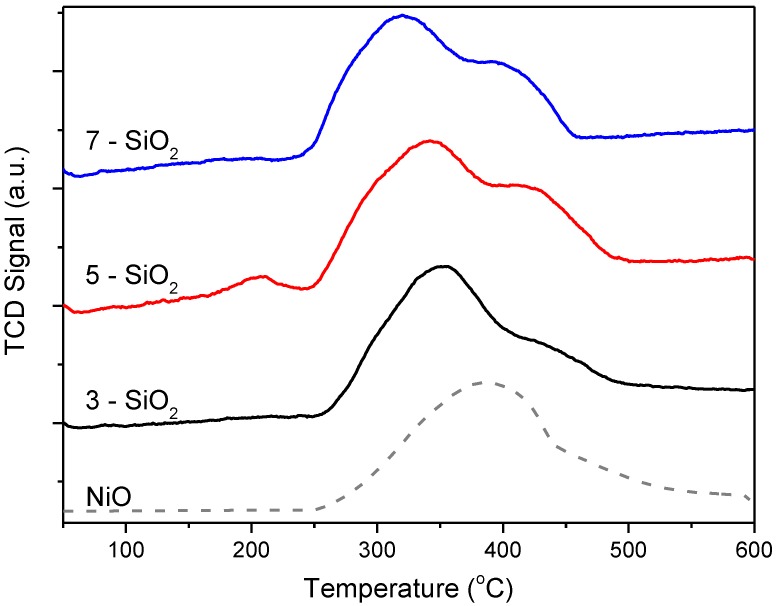
H_2_-TPR results of 5% Ni on 3, 5 and 7-SiO_2_ and bulk NiO.

H_2_-TPR was used to assess whether there was an impact of varying FSP support properties on NiO-support interaction with the reduction profiles shown in Figure 4. The H_2_-TPR profile for NiO powder (dashed line) was included for comparison. All reduction profiles show one main reduction peak (at approximately 320–350 °C) coupled with a broad, higher temperature shoulder at greater than 400 °C. The shoulder occurs at a similar temperature to the bulk NiO and is therefore assigned to NiO deposits weakly interacting with the silica support. It follows that the more significant, lower temperature peak then corresponds to NiO deposits with stronger metal-support interaction. When comparing the profiles between 3, 5 and 7-SiO_2_ it is clear that there are some differences in metal-support interaction, even though all samples show the presence of these two Ni species, in varying proportions. 3-SiO_2_ shows the lowest relative quantity of bulk NiO, which is consistent with the TEM particle size distributions. 5-SiO_2_, on the other hand, shows a significantly higher amount of bulk NiO. An additional slight hump present at around 200 °C is evident for 5-SiO_2_ which is tentatively attributed to trace Ni_2_O_3_ [26,27], however, no peaks were evident for this particular species in XRD (vide infra) and the peak is very small thus the quantity is seemingly insignificant. Compared to 5-SiO_2_, 7-SiO_2_ shows relatively smaller quantities of bulk NiO (represented by the smaller high temperature shoulder) as well as a narrower peak. This is consistent with the TEM micrographs showing on average a smaller size as well as a narrower deposit size distribution.

**Figure 5:**
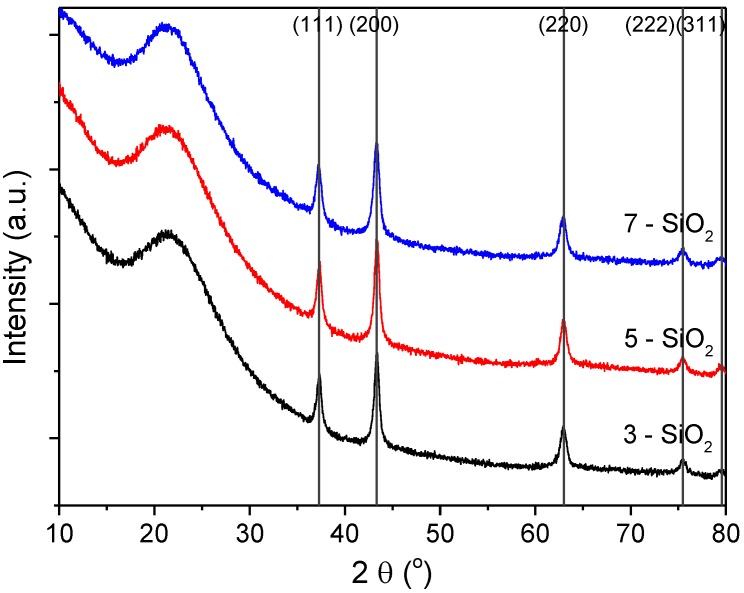
XRD patterns of 5% Ni on 3, 5 and 7-SiO_2_.

XRD was used to understand the difference in NiO crystallinity for the 5% Ni loaded on 3, 5 and 7-SiO_2_ (Figure 5). All profiles show similar patterns with peaks at 2θ values of 38, 44, 63, 76 and 79° corresponding to NiO(111), (200), (220), (222) and (311) planes, respectively. All XRD patterns also show a broad hump and tailing centered at around 23° which is characteristic of non-crystalline silica [28,29,30]. The Scherrer equation was used to estimate NiO crystal size with the results displayed in Table 1. The crystal size estimates show similar values between the differing silica supports, with a slight change in size depending on support synthesis conditions. The crystal sizes, consistent with TEM and TPR results, show a slight increase in the following order 3 < 7 < 5-SiO_2_. When comparing the average NiO deposit size determined by TEM (22–26 nm) to the crystal size from XRD (~13 nm) it can be concluded that NiO deposits are multi-crystalline for all samples. Typically H_2_ or CO chemisorption would be utilised to determine Ni dispersion. In this case, the relatively low Ni content and dispersion relative to the signal-to-noise ratio of the analysis made reliably determining the dispersion problematic. For this study, the XRD, H_2_-TPR and TEM results are sufficient in showing the impact of the differing silica support on the nature of the Ni species present.

To ensure that the impregnated samples all had consistent Ni loadings, ICP-OES was utilised with the results being displayed in Table 1. All samples show equivalent Ni loadings, thus the differences in Ni sizes do not arise from variations in Ni loading.

### 2.2. Discussion of Ni-SiO_2_ Properties

As was expected, increasing the FSP precursor feed rate decreased the SiO_2_ specific surface area over the range of 240–340 m^2^/g. As the SiO_2_ specific surface area increased it was anticipated that NiO deposit size would then decrease (*i.e.*, dispersion would increase) although this was not found to be the case. Instead, the average NiO deposit size increases in the following order; 3 < 7 < 5-SiO_2_. While the silica specific surface area was a significant factor which governed NiO deposit size, FTIR demonstrated that changing the precursor feed rate also impacted on the silica surface chemistry. In particular, increasing the FSP feed rate altered the ratio of H–bonded to isolated silanol groups on the silica surface as well introduced residual carbon species. Recent research has demonstrated the importance of silica surface properties during impregnation [7,17,18]. It is well established that there are two types of silanol groups on silica surfaces; H–bonded Si–OH and isolated (non H–bonded) Si–OH [14,15,20]. As mentioned earlier, upon impregnating SiO_2_ with Ag it was found that varying silanol groups by calcination significantly altered the active site (Ag) dispersion and metal-support interaction [17]. It has also been reported by others that modifying the surface of a silica support (by ethylene glycol pre-treatment) resulted in more dispersed Ni species giving greater activity for the DRM [18]. The studies concluded that during impregnation the Ni^2+^ interacts preferentially with H–bonded –OH groups which results in larger Ni species. Additionally, Lv *et al.*, postulated that residual C–H groups and a reduction in total surface hydroxyl groups (comparative to Si–O–Si) contributed to increased Ni dispersion [18]. It is difficult to ascertain whether the increase in Ni dispersion arises from the variation of silanol groups on the silica surface or introduction of residual C–H groups or a combination of the two. However is clear is that the difference in surface properties of the FSP-prepared silica has an impact on the dispersion and Ni-support interaction, although to a lesser extent than the impact of varying surface area. These findings demonstrate FSP is suitable for producing silica as a support for NiO catalysts with synthesis conditions able to be effectively tuned.

### 2.3. Activity Results

**Figure 6:**
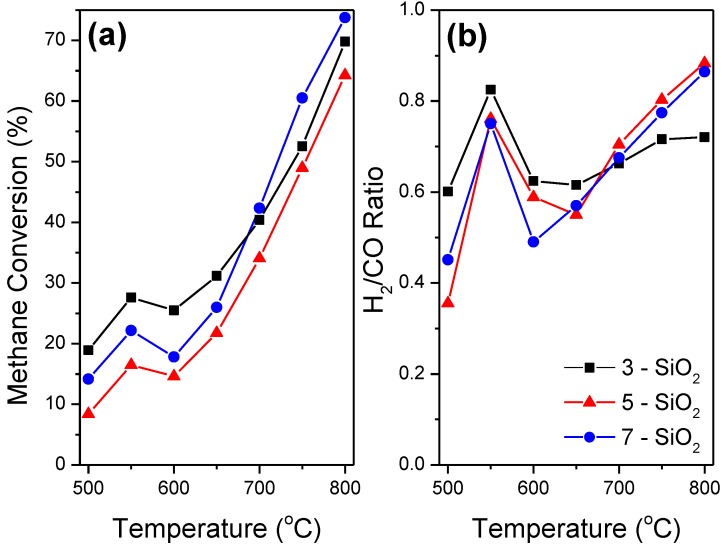
Methane light-off curves (**a**) and accompanying H_2_/CO production ratio (**b**) during dry reforming of methane over 5% Ni supported by 3, 5 and 7-SiO_2_. Conditions: WHSV = 144 L/(h·g_cat_), catalyst loading = 0.025 g, N_2_/CH_4_/CO_2_ = 1:1:1.

The impact of FSP SiO_2_ support precursor feed rate on the performance of 5% Ni-SiO_2_ for the DRM was evaluated using two approaches; (1) light-off curves over the range 500–800 °C and (2) 24 h stability tests at 700 °C. Figure 6 shows CH_4_ conversion along with the H_2_/CO ratio of each sample with respect to temperature for the light-off profiles. The H_2_/CO profiles are of most importance when considering catalysis for the DRM as they provide vital insight into the side reactions occurring within the system. The Boudouard reaction (Equation (2)) at lower temperatures (<650 °C) and methane decomposition (Equation (3)) at higher temperatures (>650 °C) result in carbon formation as well as an increase in H_2_/CO ratio. The reverse water gas shift (RWGS) reaction (Equation (4)) results in a decrease in H_2_/CO ratio, and increases in terms of thermodynamic favourability with increasing temperature [8]. A ratio of one indicates a balance of side reactions occurring within the system.

(2) $$2CO\to CO_{2}+C$$

(3) $$CH_{4}\to C+2H_{2}$$

(4) $$H_{2}+CO_{2}\to CO+H_{2}O$$

The governing trends evident from the light-off curves (Figure 6) are consistent with the thermodynamics of the side reactions within the DRM system. With the exception of temperature region 550–600 °C, all samples show an overall increase in CH_4_ conversion and H_2_/CO ratio conversion as temperature increases. CO_2_ consumption (not shown) exhibited a similar profile to the CH_4_ light of curve except it remained approximately 10%–20% higher at all temperatures. The CH_4_ conversion and H_2_/CO ratio plateau in the 600–650 °C range representing a transition between side reaction dominance. At this temperature, the side reactions occurring in the system are at a thermodynamic minimum. Specifically the Boudouard reaction becomes thermodynamically unfavourable whilst both methane decomposition along with the RWGS reaction increases in likelihood with increasing temperature. In addition, the higher CO_2_ conversion relative to CH_4_ conversion at all temperatures provides further evidence the RWGS reaction and/or CO_2_ decomposition are occurring.

5-SiO_2_ shows the lowest CH_4_ conversion over the entire temperature range which can be attributed to the larger Ni deposit size. A larger deposit size means less surface area of active sites per mass of Ni present which intrinsically results in a less active catalyst. 5-SiO_2_ also has the highest H_2_/CO ratio at higher temperature, showing the most significant increase in the 650–800 °C temperature range. H_2_-TPR, supported by TEM and XRD, show that 5-SiO_2_ has the most bulk NiO present. Bulk NiO with minimal metal-support interaction has a tendency to promote methane decomposition [6,31] and therefore will result in higher carbon formation at higher temperatures. Although the high H_2_/CO ratio implies that 5-SiO_2_ will produce the largest quantity of carbon from methane decomposition, all samples contained approximately the same quantity of carbon present after reaction. TGA indicated that the quantity of formed carbon during the DRM was 56.4, 56.6 and 57.7% for the 3, 5 and 7-SiO_2_ supports, respectively. However, the relatively higher H_2_/CO ratio at lower temperatures exhibited by 3-SiO_2_ coupled with the lower H_2_/CO ratio at higher temperature imply that there are differing dominant side reactions within the system despite the equivalent carbon formation. 3-SiO_2_ outperforms all samples at lower temperatures however, above 700 °C 7-SiO_2_ is the most active. This indicates that 3-SiO_2_ has deactivated to a greater extent at lower temperatures. The lower H_2_/CO ratio at higher temperatures and equivalent carbon formation over the full temperature range implies that 3-SiO_2_ is more prone to carbon formation, and thus elevated deactivation, through the Boudouard reaction. The implication from the results is that 5-SiO_2_ is more susceptible to carbon formation via methane decomposition whilst 3-SiO_2_ is more inclined to form carbon through the Boudouard reaction. 7-SiO_2_ seems to lie in between the 3 and 5-SiO_2_ in terms of the prominence of side reactions.

**Figure 7:**
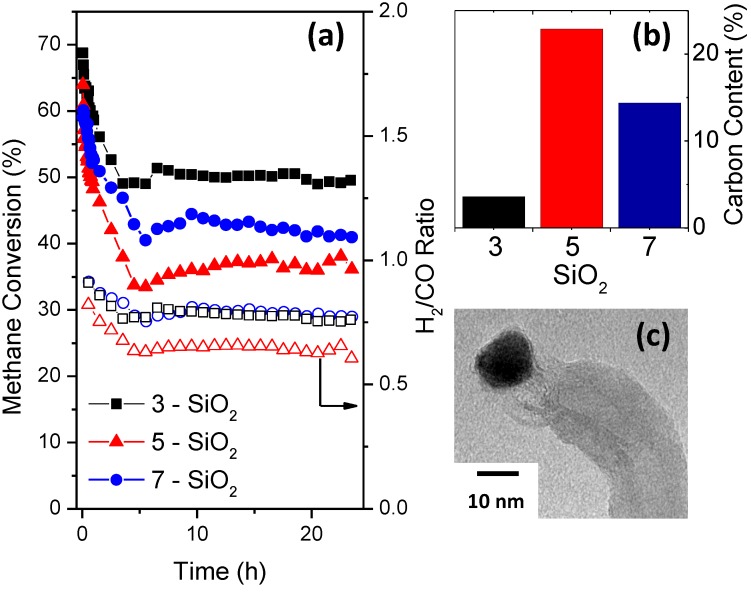
Twenty four hour stability tests with methane conversion (**a**) and accompanying H_2_/CO production ratio during dry reforming of methane over 5% Ni supported by 3, 5 and 7-SiO_2_ with (**b**) showing carbon formation determined by TGA and (**c**) TEM of spent 3-SiO_2_. Conditions: WHSV = 144 L/(h·g_cat_), catalyst loading = 0.025 g, N_2_/CH_4_/CO_2_ = 1:1:1 at a temperature of 700 °C.

The stability of the prepared catalysts was assessed by 24 h stability tests run at 700 °C in order to minimise the impact of the Boudouard reaction (Figure 7). The results indicate that, as expected, 3-SiO_2_ facilitates the highest methane conversion. The silica shows approximately 30% loss in activity over the 24 h period, with the most significant drop evident within the first 5 h. The carbon formation is significantly lower than all other samples at 700 °C, which further supports the findings drawn from the light-off curves (Figure 6), namely that 3-SiO_2_ results in a reduction in methane decomposition. TEM images (Figure 7c) show the main species of carbon formed were nanotubes, typical of Ni-catalysts for the DRM [32]. The TGA curves (not shown) did not indicate significant differences in the carbon species across the different supports.

Comparatively, 5-SiO_2_ shows the greatest deactivation, losing approximately 43% of its activity, most significantly within the first 5 h. This coupled with the highest carbon formation shows that the larger quantity of bulk Ni (as well as the largest average Ni size) present on 5-SiO_2_ results in methane decomposition thus leading to the most unstable catalyst. Interestingly, whilst the deactivation and carbon formation suggest that 5-SiO_2_ has the highest quantity of methane decomposition the H_2_/CO ratio suggests there are further side reactions occurring in the system. The implication is that 5-SiO_2_ is most prone to both the RWGS reaction as well as methane decomposition. This tendency toward the RWGS reaction results in an overall lower H_2_/CO ratio, despite the evidence of methane decomposition. 7-SiO_2_ shows an intermediate behavior in terms of deactivation (31%) and carbon formation (14.4%). This follows strongly with the measured Ni size (Section 2.2).

The differences in the activity, stability and selectivity of the produced catalysts likely originate from the varying nature of the Ni deposits on the silica surface. As the silica is a relatively inert support without significant oxygen mobility and acidity/basicity, the impacts of the varying support synthesis conditions on the activity of the prepared catalysts can be attributed to the change in Ni properties. Larger Ni deposits result in decreased conversion and an increase in carbon formation. It follows that the smaller the Ni deposits, the larger the available surface area of Ni (per mass of Ni present) and thus the highest conversion. A similar effect has been found by others for Ni-SiO_2_ samples for the DRM at 500 °C, proving that catalytic activity decreased exponentially with increasing particle size in the range of ~1.6–7.3 nm [33]. Their results demonstrated the correlation between surface Ni atoms and sample activity with the turn over frequencies (per mole of surface Ni) being independent of Ni size [33]. Additionally, larger NiO, with minimal metal-support interaction has a greater tendency to form carbon through methane decomposition (Equation (3)) [6,31]. Research has suggested the size limit for carbon filaments is <10 nm, and as in this work the NiO deposit sizes exceed this limit, the formation of carbon filaments is feasible [33,34]. For the smaller Ni deposits it is evident that at lower temperatures (<650 °C), the Boudouard reaction dominates carbon formation and results in deactivation. Additionally, Baudouin *et al.*, proved the extent to which the RWGS occurs is highly dependent on conversion (and thus particle size). Essentially they concluded that larger Ni results in a greater extent of the RWGS occurring due to the kinetics of the initial dissociation [33].

## 3. Experimental Section

### 3.1. Silica Synthesis: Flame Spray Pyrolysis

A FSP reactor, described elsewhere [4], was utilised to synthesise SiO_2_ at varying precursor feed rates. The precursor solution, 1.26 M hexamethyl disiloxane (Sigma-Aldrich, >98.5%, St Louis, MO, USA) in absolute ethanol, was prepared in a glove box to prevent hydrolysis from atmospheric moisture. The precursor was fed to the flame at 3, 5 and 7 mL/min using a syringe pump. The feed was dispersed using an oxygen (Coregas, Yennora, Australia, >99.9%) feed of 5 L/min with the pressure drop across the nozzle being maintained at 150 kPa for all samples. The precursor was ignited and the flame maintained by a supporting, pre-mixed oxygen/methane flame consisting of 3.2 L/min of oxygen and 1.5 L/min methane (Coregas, >99.95%). An additional oxygen flow of 5 L/min was used as a sheath gas. The synthesised silica was collected on a glass fibre filter with the use of a vacuum pump (Whatman, GE Healthcare Life Sciences, Buckinghamshire, UK). The fuel equivalence (Φ) ratio was calculated as the ratio of actual fuel (HMDSO, ethanol and methane) to oxidant to the stoichiometric fuel to oxidant ratio within the flame [3].

### 3.2. Impregnation and Calcination

The silica produced at different feed rates was impregnated with 5 wt % Ni. Aqueous nickel nitrate (0.1 M Ni(NO_3_)_2_·6H_2_O, Asia Pacific Specialty Chemicals Ltd. >98.5%, Sydney, Australia) precursor was added to the silica and mixed for 3 h before drying at 100 °C for 16 h. After drying the samples were finely ground using a mortar and pestle before calcination. To calcine, the samples were loaded in a fixed bed glass reactor (100 mm inner diameter) and heated under 60 mL/min air (Coregas Dry Compressed Air) at 5 °C/min to 450 °C where they were held for 4 h.

### 3.3. Catalyst Characterisation

N_2_ physisorption isotherms and BET specific surface areas were obtained at −196 °C on a Micromeritics Tristar 3000 (Micromeritics, Norcross, GA, USA) with the samples being degassed for 3 h at 150 °C under vacuum prior to measurement. FTIR spectra were recorded using a Spotlight 400 FTIR (PerkinElmer, Waltham, MA, USA) at room temperature from 600–4000 cm^−1^ with an instrumental resolution of 4 cm^−1^ and each spectra being taken as the average of 8 scans. For transmission electron microscopy (TEM) the samples were ultrasonically dispersed in methanol and then dropped onto a carbon-coated copper grid. The micrographs were recorded using a Philips CM200 FEG TEM at 200 kV (CM200 TEM, Philips Co., Amsterdam, The Netherlands). For NiO particle size distribution at least 300 particles were measured. The spread of the data was calculated by sample standard deviation (σ). XRD was used to determine NiO crystallite size. The sample was scanned with CuK_α1_ radiation (λ = 0.154 nm) at 45 kV and 40 mA from 10 to 80° with a 0.026° step size using an PANalytical Xpert Multipurpose X-ray Diffraction System (Philips Co., Eindhoven, The Netherlands). The NiO crystal sizes were estimated from the Scherrer equation with a shape factor, K, of 0.9. To determine NiO reducibility H_2_-TPR was studied on a Micromeritics Autochem 2920 system with a thermal conductivity detector (TCD, Micromeritics, Norcross, GA, USA). Approximately 50 mg of sample was loaded into a quartz U-tube sample holder. The sample was pre-treated at 150 °C (10 °C/min) for 0.5 h under Ar (Coregas Argon, >99.999%) and then cooled to 50 °C before being heated to 900 °C (5 °C/min) under 5% H_2_ in Ar (10% H_2_ in Ar diluted by Ar) at 20 mL/min. Bulk NiO was produced as a reducibility comparison by calcining nickel nitrate (Ni(NO_3_)_2_·6H_2_O) for 4 h at 450 °C (5 °C/min ramp rate). To determine the actual Ni loading inductively-coupled plasma optical emission spectrometry (ICP-OES, PerkinElmer, Waltham, MA, USA) was used. Samples were partially digested by heating to 90 °C in aqua-regia before being diluted and analysed using a PerkinElmer OPTIMA 7300 system.

TGA was used to determine the carbon content on spent samples. The sample (approximately 2 mg) was loaded into a Pt sample holder and heated to 800 °C at 20 °C/min in air (Coregas Dry Compressed Air, 100%) on a TA instruments TGA Q5000 (TA instruments, New Castle, DE, USA). To account for water desorption from the catalyst surface, only the mass lost between 300–800 °C was selected.

### 3.4. Catalytic Activity Tests

Catalytic activity was evaluated in two ways; light-off profiles and 24 h stability tests. Tests were completed at atmospheric pressure with 25 mg of sample being diluted with 25 mg of neat silica (Fluka, Sigma-Aldrich, Silica Gel 40, surface area of 522 m^2^/g). Blank tests with diluent silica showed negligible methane conversion at 800 °C. The diluted sample was loaded into a quartz reactor (6 mm ID, 300 mm long) between two plugs of quartz wool with a thermocouple being positioned within the reactor, directly above the catalyst bed. Samples were reduced in-situ prior to activity tests. Samples were heated, at 1 °C/min, to 350 °C and held for 10 h under 60 mL/min of 50% H_2_ in Ar (Coregas Hydrogen, >99.99%; Coregas Argon, >99.992%). Samples were then cooled to room temperature under 60 mL/min Ar.

For the light-off profiles the reduced samples were heated under N_2_ (60 mL/min; Coregas Nitrogen, >99.999%) to 500 °C (5 °C/min). After holding for 0.5 h, the reactants, in the form of a 1:1:1 of CH_4_/CO_2_/N_2_ (Coregas Methane, >99.95%; Coregas Carbon Dioxide, >99.99%), were introduced at a weight hourly space velocity (WHSV) of 144 L/(h·g_cat_). The system was held at 500 °C for 0.5 h and then heated to 800 °C in 50 °C increments (5 °C/min) with a 0.5 h hold at each step. For the 24 h stability tests the reduced and cooled catalysts were heated under 60 mL/min N_2_ (Coregas Nitrogen, >99.999%) to 700 °C (5 °C/min) and held for 0.25 h. A cold trap with ice was located between the reactor outlet and GC inlets to remove any moisture from NiO reduction and the reverse water gas shift reaction. The reactant gases were then introduced at the same ratio and WHSV as above and the system retained at 700 °C for 24 h. The produced gases were analysed by two online gas chromatographs; a Shimadzu 8A with a TCD and Molecular Sieve 13X column for H_2_ and N_2_ detection and a Shimadzu 2010 with a flame ionisation detector (FID) and HP/Plot Q column for CH_4_, CO_2_ and CO detection. Methane conversion and H_2_/CO ratio were calculated from Equations (5) and (6), respectively.

(5) $$x_{CH_{4}}=\frac{{\left[CH_{4}\right]}_{in}-{\left[CH_{4}\right]}_{out}}{{\left[CH_{4}\right]}_{out}}\;\times 100$$

(6) $$\frac{H_{2}}{CO}=\frac{{\left[H_{2}\right]}_{out}}{{\left[CO\right]}_{out}}$$

## 4. Conclusions

These findings demonstrate the capacity for FSP to produce silica as a support for Ni-based catalysts for the DRM. The synthesis of silica with controllable size (and thus surface area) was achievable by simply varying the feed rate of precursor to the flame. By altering the flame synthesis conditions, the surface chemistry of the produced silica was significantly impacted. By increasing the precursor feed rate (without altering the oxygen feed), the fuel equivalence ratio within the flame was increased which in turn resulted in the introduction of residual, non-combusted carbon species as well as altered the silanol groups on the silica surface. The lower feed rate (3 mL/min) resulted in the highest silica surface area (340 m^2^/g) which facilitated the smallest Ni size and thus most active and stable catalyst at higher reaction temperatures. The highest silica feed rate (7 mL/min) introduced residual carbon-based species on the silica surface and therefore, although having the lowest surface area, was able to obtain relatively high Ni dispersion (comparative to the 5 mL/min sample). This in turn resulted in an increase in activity for the DRM.

Our results have successfully illustrated the ability for FSP to synthesise silica as a support for Ni catalysts for the DRM. Moreover, we have shown the ability to tune the properties of FSP prepared silica to be able to control Ni properties.
